# The Impact of COVID-19 on Older Adults’ Perceptions of Virtual Care: Qualitative Study

**DOI:** 10.2196/38546

**Published:** 2022-10-20

**Authors:** Lama Abdallah, Paul Stolee, Kimberly J Lopez, Alexandra Whate, Jennifer Boger, Catherine Tong

**Affiliations:** 1 School of Public Health Sciences University of Waterloo Waterloo, ON Canada; 2 Department of Recreation and Leisure University of Waterloo Waterloo, ON Canada; 3 Department of Systems Design Engineering University of Waterloo Waterloo, ON Canada

**Keywords:** virtual care, older adult, qualitative, COVID-19, elderly population, aging, telehealth, digital care, technology usability, patient perspective, technology access

## Abstract

**Background:**

In response to the COVID-19 pandemic, older adults worldwide have increasingly received health care virtually, and health care organizations and professional bodies have indicated that virtual care is “here to stay.” As older adults are the highest users of the health care system, virtual care implementation can have a significant impact on them and may pose a need for additional support.

**Objective:**

This research aims to understand older adults’ perspectives and experiences of virtual care during the pandemic.

**Methods:**

As part of a larger study on older adults’ technology use during the pandemic, we conducted semistructured interviews with 20 diverse older Canadians (mean age 76.9 years, SD 6.5) at 2 points: summer of 2020 and winter/early spring of 2021. Participants were asked about their technology skills, experiences with virtual appointments, and perspectives on this type of care delivery. Interviews were digitally recorded and transcribed. A combination of team-based and framework analyses was used to interpret the data.

**Results:**

Participants described their experiences with both in-person and virtual care during the pandemic, including issues with accessing care and long gaps between appointments. Overall, participants were generally satisfied with the virtual care they received during the pandemic. Participants described the benefits of virtual care (eg, increased convenience, efficiency, and safety), the limitations of virtual care (eg, need for physical examination and touch, lack of nonverbal communication, difficulties using technology, and systemic barriers in access), and their perspectives on the future of virtual care. Half of our participants preferred a return to in-person care after the COVID-19 pandemic, while the other half preferred a combination of in-person and virtual services. Many participants who preferred to access in-person services were not opposed to virtual care options, as needed; however, they wanted virtual care as an option alongside in-person care. Participants emphasized a need for training and support to be meaningfully implemented to support both older adults and providers in using virtual care.

**Conclusions:**

Overall, our research identified both perceived benefits and perceived limitations of virtual care, and older adult participants emphasized their wish for a hybrid model of virtual care, in which virtual care is viewed as an addendum, not a replacement for in-person care. We recognize the limitations of our sample (small, not representative of all older Canadians, and more likely to use technology); this body of literature would greatly benefit from more research with older adults who do not/cannot use technology to receive care. Findings from this study can be mobilized as part of broader efforts to support older patients and providers engaged in virtual and in-person care, particularly post–COVID-19.

## Introduction

As a result of efforts to limit the spread of the virus that might occur through in-person appointments, the COVID-19 pandemic accelerated the shift to virtual health care. Virtual health care, subsequently, was widely adopted across Canada and beyond [1-5]. Simultaneously, policies at the institutional, national, and international levels flexed to accommodate recommendations on the use of virtual care within existing health care models [6,7]. Virtual care can be defined as “any interaction between patients and/or members of their circle of care, occurring remotely, using any forms of communication or information technologies, with the aim of facilitating or maximizing the quality and effectiveness of patient care” [8]. Virtual care is not limited to a particular technology or platform (eg, it can include the telephone) and is often used interchangeably with “telemedicine” or “eHealth” [8,9]. Prior to the COVID-19 pandemic, virtual care activities, although possible, were not common in Canada [10,11]. Although the COVID-19 pandemic sparked a dramatic increase in virtual care in Canada [1,2] and worldwide [4], questions remain about the quality and role of virtual care in practice [6,12], particularly with older patient populations.

Although Bhatia et al [1] found that older patients were the highest users of virtual care during the pandemic, Senderovich and Wignarajah [13] expressed concerns about the maintenance of the therapeutic alliance between physicians and older patients receiving virtual care (a therapeutic alliance being a patient-doctor relationship that supports positive health outcomes). Prepandemic research in the United Kingdom by Hammersley et al [12] found that older patients were less likely to choose virtual care than were younger patients. The experience of older patients with virtual care is thus of continued interest, both during and after the pandemic. Despite common misconceptions about older adults and technology, a national survey conducted in July 2020 found that 72% of older Canadians feel confident about their ability to use existing technologies, such as smartphones or video calls [14]. In the 3 months prior to the July 2020 survey, 52% of older Canadians accessed virtual care and 79% were satisfied with the virtual care received [14]; the bulk of the virtual care they received was over the telephone. Although studies have investigated the use of virtual care with older adults before (eg, [12,15-18]) and during (eg, [19,20]) the COVID-19 pandemic, this evidence is largely quantitative; there is a lack of qualitative data that reflect the perspectives and experiences of older Canadians accessing virtual care throughout the pandemic. Lopez et al’s [21] analysis of older adults’ use of technology during the pandemic found a notable increase, including broader adoption of videoconferencing software/video calls. Teti et al [22] emphasize the importance of reflecting qualitative data throughout the COVID-19 pandemic to understand how COVID-19 impacts populations as a social event as well as a medical pandemic. Qualitative approaches play a vital role in understanding social responses to pandemics, as they allow us to understand the lived experiences of those who are disproportionately impacted, including older adults [22].

The aim of this study was to use a systematic qualitative study to understand how older adults experienced virtual care during the pandemic and to include their perspectives on virtual care as an alternative or supplement to in-person care. Organizations, such as the Canadian Medical Association (CMA), have indicated that virtual care is “here to stay,” even if/when no longer necessitated by the pandemic. If virtual care is indeed here to stay, our interviews with older adults will contribute to broader discussions on how and when to use virtual care in a manner that reflects their experiences, wishes, and perspectives.

## Methods

### Study Design

This research is part of a larger study [21] in which we used a longitudinal qualitative study [23] approach to listen to older adults speak about their social connections and experiences of digital connectivity early (summer of 2020) and later (winter and early spring of 2021) in the COVID-19 pandemic. Our research team is situated in Ontario, Canada, and eligible participants included any older Canadian (aged 65 years or more) able to complete an English-language telephone/video interview and provide informed consent.

### Ethical Considerations

We received ethics clearance from the University of Waterloo’s Office of Research Ethics (ORE #42265).

### Recruitment

A purposive sampling strategy [24] was used to recruit a diverse sample of older adults (eg, rural/urban; community/assisted living; diverse abilities, socioeconomic profiles, genders, and ethnicities). Recruitment during the beginning of a global pandemic that was disproportionately impacting older adults was challenging, and we used several recruitment approaches to access diverse older adults. We recruited using social media (eg, Twitter), emails to large established groups with older adult members (blinded for review), telephone calls to older adults within our personal networks (ie, asking our personal contacts to share study materials within their networks), and promotion of our study via teleconferences with older adult participants. In total, 20 older adults completed the baseline in-depth interviews in the spring of 2020, which coincided with the first wave of COVID-19 in Canada. In the spring of 2021, follow-up interviews were conducted with 12 (60%) participants from the baseline sample, coinciding with the second wave of COVID-19 in Canada. Of the 12 participants, 8 (67%) did not participate in the follow-up interviews because of death (n=1, 12.5%), they could not be reached (n=3, 37.5%), or they declined to participate in a second interview (n=4, 50%). Recruitment for follow-up interviews coincided with a particularly challenging period of the pandemic (ie, stringent lockdowns; rising case counts and deaths, especially among older adults; and the darker, bleaker winter months); 3 (75%) of the 4 participants who declined to follow-up specifically expressed that this was because of the challenging period and timing. Participant characteristics are summarized in Table 1.

**Table 1:** Participant characteristics.

Characteristics		Participants	
		Baseline (N=20)	Follow-up (N=12)
**Gender, n (%)**			
	Women	14 (70)	8 (67)
	Men	6 (30)	4 (33)
**Age (years)**			
	Range	66-94	66-94
	Mean (SD)	76.9 (6.5)	77.3 (6.8)
**Living arrangement, n (%)**			
	Alone	7 (35)	3 (25)
	With spouse	10 (50)	6 (50)
	With adult child	1 (5)	1 (8)
	Assisted living facility	2 (10)	2 (17)
**Community type, n (%)**			
	Urban	11 (55)	6 (50)
	Suburban	1 (5)	1 (8)
	Rural	8 (40)	5 (42)
**Province, n (%)**			
	Ontario	17 (85)	10 (83)
	Alberta	3 (15)	2 (17)
**Race, n (%)**			
	White	16 (80)	10 (83)
	BIPOC^a^	4 (20)	2 (17)

^a^BIPOC: Black, Indigenous, and people of color.

### Data Collection

All interviews were conducted over the telephone or via videoconferencing software. Baseline interviews lasted an average of 53 minutes (minimum 24 minutes; maximum 74 minutes); follow-up interviews lasted an average of 60 minutes (minimum 21 minutes; maximum 112 minutes). The interview questions (Multimedia Appendix 1) were developed in consultation with older adults from our “Seniors Helping as Research Partners” group and informed by our interdisciplinary research team, which includes experts in systems design engineering for older adults, recreational therapy, social gerontology, and designing health care systems for older patients. The first two-thirds of the interview focused on participants’ use of and access to technology, comfort with technology, etc, and the final third of the interview focused specifically on virtual health care. Interviews and analytic debriefs were digitally recorded, run through otter.ai transcription, and then cleaned and anonymized by research assistants using protocols established by our team. Anonymizing the transcripts included the assignment of a pseudonym for each participant. Additional details about the overarching study, recruitment, and data collection may be found here [21].

### Data Analysis and Strategies

Our team-based analysis (ie, multiple members of the research team, drawing on different disciplinary perspectives to collectively analyzing the data; see Guest and MacQueen [25]) process used a framework analysis approach [26] that included the following steps:

- Step 1 (familiarization): Each transcript was read by 1 of 3 coauthors (LA, CT, and AW), who were the same coauthors who conducted the interviews.
- Step 2 (development of a coding framework): All coauthors used the initial read of the data, field notes, and debriefs to develop an initial set of thematic codes.
- Step 3 (indexing and charting): Three coauthors, (LA, CT, and AW) engaged in line-by-line coding [27].
- Step 4 (summarizing and synthesizing): The coding structure was further refined through team analysis meetings and shared coding memos to consolidate the most salient themes presented later.

Rigor strategies included reflexive memoing, an audit trail within NVivo (QSR International) [28], and team-based examination of the data and each step of the analysis [25]. We also reviewed our findings and interim analysis with 4 participants (ie, member checking and reviewing our interpretations of the data) via an online focus group that were recorded and transcribed to inform the analysis.

## Results

### Participant Details

We interviewed a total of 20 patients at baseline (14, 70%, women; 6, 30%, men) and 12 at follow-up (8, 67%, women; 4, 33%, men). Ages ranged from 66 to 94 years, with an average of 76.9 (SD 6.5) years. Most patients lived alone or with a spouse, in urban or rural communities of Ontario and Alberta. Of the 20 patients, 4 (20%) were Black, Indigenous, or people of color (BIPOC) and the remainder (n=16, 80%) were White.

In discussing their experiences with virtual care during the pandemic, older adults broadly shared 3 high-level themes: (1) their experiences accessing health care during the pandemic, (2) their perceived benefits and limitations of virtual care, and (3) their perspectives on when virtual care is acceptable and appropriate. In the quotes presented later, the suffixes included after the patient pseudonym and biographical information (B and F) refer to baseline and follow-up interviews, respectively. Participants often shared their perspectives on virtual care in the first interview and in the second replied, “Like I said last time…”; thus, more of the presented quotes are from the baseline interviews than from the follow-up interviews. There was not a notable change in participants’ perspectives on virtual care across the 2 time points.

### Experiences With Health Care During the Pandemic

Participants described their health care experiences during the pandemic in terms of issues with accessing care, and their pandemic experiences of in-person and virtual care. Although we did not specifically probe for issues with accessing care, many participants mentioned that they had not seen their primary care providers for months; some had not contacted their providers since the start of the pandemic:

No, because I haven’t been in touch with them since well…

Richard, 76 years, male, B

Since March.

Joan, 66 years, female, B

No, I haven't seen my doctor this year.

Richard, 76 years, male, B

At baseline, Susan, aged 82 years, expressed that older adults are reluctant to access in-person care because they are weighing the risk of contracting COVID-19 against the risk of missing an appointment:

And, and the other thing is that people hesitated maybe too much sometimes to go to the hospital. Like you said, people full of coughing in an emergency room. But, you know, there are situations where people might have delayed going and they needed to go…Yeah, they're really…it's assessing the risk. Like, you know, maybe I'm better to stay home than to get COVID.

Susan, 82 years, female, B

The disruption and discontinuity of care resulting from the COVID-19 pandemic caused participants to feel fear and anxiety about the frequency and quality of care they received. However, in comparison to the risk of contracting COVID-19 when accessing in-person care, they seemed to fear the virus more than the potential complications that could result from missing so many in-person appointments.

Participants felt that they accessed in-person care less regularly than they would have prior to the pandemic. In-person care was mostly accessed for emergencies, specialist services (eg, oncology, physiotherapy), and services that could not be accessed online (eg, diagnostic imaging, blood work). When in-person care was accessed, some aspects of the care were organized virtually. At baseline, Nancy, aged 66 years, had an x-ray performed in-person, with the results of the x-ray communicated virtually:

I've had one X-ray and that's about it, I think.

Nancy, 66 years, female, B

Okay. And then you've got the results of the x-ray over the phone?

AW

That's right, yeah.

Nancy, 66 years, female, B

### Virtual Care

When asked about their experiences accessing virtual health care during the pandemic, most participants were able to discuss a time when they accessed care virtually either at baseline or follow-up. Virtual care usually involved phone calls for completing intake, scheduling, and accessing appointments; text or email messages for sending photos of health concerns; and emails/phone calls for receiving requisitions and test results. Few participants accessed virtual care in the form of video calls; most virtual care had been provided over the telephone, with some referrals or results (eg, of bloodwork) being communicated over email. In general, participants were satisfied with the virtual care they received from their family physicians. Participants also felt their relationships with regular providers were not negatively impacted by the pandemic; participants maintained their patient-doctor relationships virtually despite changes in care delivery and frequency. Participants were less comfortable with certain tasks being performed virtually, such as being prescribed a new medication, diagnosis, or meeting with a specialist for the first time.

#### Perceived Benefits of Virtual Care

When prompted about the benefits of virtual care, all participants identified at least 1 positive aspect of virtual care compared to in-person care. The most common perceived benefit was convenience, which was discussed by most participants. Other commonly cited benefits were improved safety due to the avoidance of unsafe situations associated with in-person care (eg, contracting a virus) and the efficiency of the health care provider. Table 2 summarizes the perceived benefits of virtual care identified by participants.

**Table 2:** Summary of the perceived benefits of virtual care use for older adults.

Benefit	Description	Quote
Convenience	Virtual care is more convenient than in-person care due to the ease of communication, including the capacity to communicate by a phone or video call instead of making a trip to the provider’s office; the ability to send and receive documentation, including referrals, requisitions, and test results virtually; and the time that is saved when not sitting in waiting rooms.	“…something like a requisition for an x-ray, that certainly…I didn't have to worry about handling the requisition. It was just transferred electronically. And, and when I appeared at the x-ray lab, I just, it was all already there. That was convenient.” [Nancy, 66 years, female, B^a^] “Yes. And then you don’t have to go to see her or him. You can just use your phone. And then that’s easier.” [Lily, 77 years, female, B]
Safety	Virtual care makes it easier to avoid unsafe situations for older adults, such as driving during the winter/bad weather, making unnecessary trips, and interacting with other people on public transit or in the waiting room who may have a communicable disease.	“It means that you, that people, elderly people particularly, don't have to leave their home, which in some…Because, sometimes, if one person is really ill, and they need somebody to go with them and then it, you wonder sometimes if you're hurting your health more by going than by staying home sort of thing.” [Susan, 82 years, female, B] “And as you, as you get older, and now we go back to winter, you know, you really don't want to drive in winter, hence the reason why we go away for 3 months…Uh, you know you're risking, as I'm saying, you're getting older, you're not as quick on the draw as far as driving is concerned and so on. So, you're risking somebody's life really going in just to do that. Whereas if you can get it on the emailer…then it makes more sense” [Katherine, 74 years, female, B]
Efficiency of health care provider	With the introduction of virtual care, providers can improve the efficiency of their practices. A couple of participants also highlighted that sharing information and engaging in appointments with larger care teams can be easier with virtual care.	“And I think probably we're going to end up going that way a little bit. It does free up doctors to deal with bigger problems, maybe. And I, I, as I say, I have not used it. So, I really don't have any personal experience about it. But my understanding from people that I know that have phoned them, the doctor generally gets back to them ASAP. And, my one daughter has a doctor friend and, the doctor seems not to be as busy.” [Shirley, 77 years, female, B] “I think, well, especially if they were going to use Zoom or something like that, if you wanted to talk to the doctor face to face and actually see her, I think that would be great if they use Zoom rather than having us go in every time for something simple… It opens the door for them to take, as I said before, to take people in that really, really, really need to see the doctor. It saves us time, saves her time. I think there's a lot of pros.” [Katherine, 74 years, female, B]

^a^B: baseline.

#### Perceived Limitations of Virtual Care

Participants identified many aspects of virtual care that they perceived to be more challenging or less effective compared to in-person care. Many identified limitations they believed would impact others (eg, the challenges less tech-savvy older adults would face while accessing and using technology, lack of access to technology for all older adults) but maintained that virtual care was ideal for themselves and had few negative aspects. The most cited limitations of virtual care describe a lack of nonverbal communication (eg, facial expressions and body language) and limited opportunities for physical examination. Other limitations included challenges with older adults accessing and using technology, challenges with patients’ and doctors’ ability to express themselves verbally (eg, in telephone-only appointments), negative impacts on care coordination and continuity, and the potential exacerbation of the social isolation of older adults (ie, for some isolated older adults, in-person visits to primary care are an essential piece of their limited social lives). One participant expressed concerns about accommodations for older adults who require language interpretation services while accessing health care. Participants’ perceived limitations are presented in Table 3.

Participants highlighted an important caveat to our interpretation of the data: a benefit of virtual care for one older adult can be a limitation of virtual care for another. For example, although one person may appreciate the efficiency of a virtual care appointment, another may deeply miss the interpersonal and social interactions that accompany an in-person visit.

**Table 3:** Summary of the perceived limitations of virtual care use for older adults.

Limitation	Description	Quote
Nonverbal communication and body language	Usual forms of virtual care (ie, phone calls and video calls) eliminate nonverbal communication, including facial expressions and body language.	“…you do miss some of the eye contact and the body language, and I make the point that when people communicate, they often talk about the words only being about 7%, the tone being 38% of the…body language being 55%, so email or a phone, you might get the tone but you don't get the body language and that's, and, sometimes that's very important. I know, how many times that I noticed body language, that I would ask another question, and bingo, the real problem would come out, where it wouldn't have come up if you hadn't been able to observe the body language” [James, 75 years, male, B^a^]
Technology	Virtual care can be difficult to access for older adults who either do not have access to sufficient technology at home or do not know how to use the technology they have to engage in virtual care.	“I worry about that. And for people who don’t have access! I mean everybody doesn’t have a computer at home, or they have a computer, but they barely don’t know how to use it. I talk to people, and they say, well, you know, my son will help me, my daughter will help me, my grandkids will help me. But other than that, they don’t know how…they don’t use it. Or they might use it just for…phone conversation…you know, for e-email kind of stuff, and that’s it. And so, they don’t get it…they don’t get to use. They don’t have real access, and now…the library is being closed now. People have even less access.” [Helen, 77 years, female, B]
Verbal communication	Participants are concerned about the ability of both patients and doctors to express themselves in virtual visits. They mentioned that people with cognitive or hearing difficulties may find accessing virtual care especially difficult due to the challenges of understanding.	“Maybe I didn't explain it well enough to them. I'm not a nurse, you know, and I just know how it feels to me, I probably don't have an experience and that, whatever is happening to me, this time when I would call, you know. So, it might be my terminology, my reporting might not be as good as they might need.” [Sharon, 82 years, female, B] “Yes, and if they have hearing problems, that might be, a deterrent too because…or cognitive problems where they, have problems understanding” [Shirley, 77 years, female, B] “We have a tendency to save the important questions ‘til the end. That's a known fact, is that people are having a physical or whatever, you go through all the steps. And as they're walking out the door they say, 'Oh, by the way, I've been having chest pains’.” [Helen, 77 years, female, F^b^]
Physical examination	Although participants believed that many health issues can be successfully discussed virtually, several participants also expressed concerns over the lack of physical and tactile examination during virtual appointments. The general concern behind this was that doctors would be more prone to accidentally missing something if the patient was not physically in front of them.	“I just want to say that it is very limited. Sometimes, when you have a problem and you're seeing a doctor, you want him to look, with his own eyeballs to see the actual thing. You…to see your skin, in the real thing not…not done through a camera, and you want him to poke you, you know, or feel. There's so much in an examination, that should be done tactile, as opposed to only visual. Only visual, you miss so much without the tactile attached to it.” [Richard, 76 years, male, B]
Care continuity and coordination	Participants felt that because virtual care usually meant that their provider was physically seeing them less often than they would with in-person care, the continuity of care and ability of physicians to coordinate care activities suffered.	“The cons would be, perhaps a lack of follow up sometimes, because you’re not seeing anything done. Like if I go to his office and he gives me a referral to someone or if he’s…you just don’t see that referral happening through you, you see him doing it, and sometimes that doesn’t happen as quickly as it could.” [Nancy, 66 years, female, B]
Social isolation and health care as a social experience	Health care visits can be vital social experiences for older adults. Switching to a virtual format removes much of the social activity and personality from appointments.	“I think if I really needed to see the doctor, she would let me go in and talk to her. And I think I still need that if I do need it. And that would be most likely for an emotional situation more than anything” [Judith, 75 years, female, F] “And also, for some people the doctor's visit is one of your social experiences. The more you live alone, like I live alone, these kinds of contacts are part of your…socialization, your contacts…like going to the library, going to the doctor, these are all things where people have contact with others. So, if you make these things more virtual, you cut back on people's contacts with the outside world.” [Helen, 77 years, female, B]

^a^B: baseline.

^b^F: follow-up.

#### Perspectives on When Virtual Care Is Acceptable and Appropriate

Subthemes related to participant perspectives on the acceptability and appropriateness of virtual care included receptiveness to virtual care in some scenarios, preferences for the future of virtual care, and the willingness of older adults to adapt to virtual care and the supports required for them to do so.

#### The Future of Virtual Care and the Preference for a Hybrid Model (Some In-Person)

When participants were asked how they would like the health care system to operate postpandemic, participants presented 2 main preferences: approximately half expressed their preference to return to a health care system that provides the majority of services in-person, while the other half preferred to retain some aspects of the COVID-19 era virtual care and reintroduce aspects of in-person care to create a hybrid system of health services.

Participants who preferred to return to an in-person model of health care were not necessarily opposed to the use of virtual care. Some agreed that, although virtual care was useful during the COVID-19 pandemic, they would prefer to access in-person care whenever possible:

Something like prescription renewals will be convenient to have them continue through the pharmacy, to my doctor, that would be very convenient. But aside of that, I'd rather see my physician in person.

Nancy, 66 years, female, B

Many participants expressed support for a hybrid health care model that includes aspects of both virtual and in-person care:

But if it went back, it went back but in a modified way, like it's not all one or all the other. It's not all phone or all office, like it could be a mix.

Patricia, 82 years, female, B

Helen, aged 77 years, expressed that although she preferred a hybrid model of care, it would need to be carefully organized and implemented to be effective:

But it needs to be carefully thought of. And I've always been hesitant about virtual care, ‘cause I don't want to see that as an instead…yes, virtual care yes, but it has to be in addendum. It has to be something in between. It's very useful to check up on something.

Helen, 77 years, female, B

Although many participants supported a future that incorporated aspects of both virtual and in-person care models, they were concerned about how this would be funded at a system level, whether doctors would find it useful or difficult to manage, and how virtual care would be organized and regulated in practice.

#### Adapting to Virtual Care

Many participants felt that older adults would be proactive in learning the technologies necessary to support themselves while accessing virtual care, as health care is viewed as a “priority” or “essential” and not an option like other technologies that might be used for entertainment, etc. However, participants felt strongly that a shift toward virtual care must include meaningful and senior-friendly training and supports that will allow older adults to learn to use the technologies required, as well as enable access to the system using technologies with which older adults feel more comfortable. Although many participants noted a need for technology training and supports for older adults, several noted that efforts aimed at improving virtual care should also be focused on training for *providers*, not just patients:

I think the…the…to take advantage of those types of situations I think technology use, I think somebody should actually encourage the GP and their registered…their nurses or their receptionist to be more proficient in these technologies. I think seniors when there's a need, they’ll do anything to learn it.

Geraldine, 72 years, female, B

Although many participants were reconciled to virtual care being a major component of their health care in the future, they saw a need for related training and support for both providers and patients.

## Discussion

### Principal Findings

We identified 3 high-level themes in our interviews with older adults about their virtual care use during the pandemic. Older adults shared (1) their experiences with virtual and in-person health care during the pandemic, (2) their thoughts on the benefits and limitations of virtual care, and (3) their opinions on when virtual care is most appropriate. Consistent with the results of the AGE-WELL [14] survey, most of the participants in our study experienced some form of virtual care access, primarily via telephone or online, with fewer participants having accessed care via video. Participants expressed reluctance to attend in-person visits during the pandemic, with in-person care accessed mainly in emergencies or for services that were not available virtually.

### Comparison With Prior Work

Prepandemic studies of virtual care (eg, [18]) have found both benefits and limitations; this was also the case for the participants in this study. Importantly, most participants felt they were able to maintain their patient-doctor relationship despite the change in the mode of care delivery, thus alleviating some of the concern raised by Senderovich and Wignarajah [13] about maintaining the quality of the therapeutic alliance. Our study participants described the convenience of virtual visits as well as increased safety, including the avoiding of unsafe travel conditions, similar to findings by Elliott et al [29]. Participants also felt virtual care would be more time efficient for the provider, but we note that some studies of virtual care have not found cost-saving benefits [30]. In contrast, our study participants recognized the lack of physical, hands-on examinations in a virtual care appointment, as was also found in studies by Breton et al [31] and Mao et al [32]. Participants also noted limitations in terms of compromises in both verbal and nonverbal communication. This is consistent with the finding of Hammersley et al [12] that there was less information sharing in virtual visits, although these authors also noted that the virtual care visits they studied revealed somewhat greater efforts toward building rapport with the patient.

Some limitations of virtual care might be mitigated if video-and-voice appointments are used. Although video appointments are not a complete substitute for a tactile examination of the body, they enable synchronous visual examination, which may help alleviate patient concerns that providers may miss something. Video calls may also enable both patients and providers to interpret nonverbal cues and facial expressions more accurately. Conversely, it may be more difficult for providers to see the patient in a video call compared to a photo of an affected area (sent via email or text) due to the wide range of devices that older adults and clinicians use, variations in connectivity or access to a reliable internet connection among patients, and compatibility between devices. In addition, video visits make additional demands on the patient, who must be able to get online and manage the technology, which may be difficult due to disability or lack of experience with technology or a stable internet connection [32,33].

### Limitations of This Study

First, our study is bound to a specific time and rooted in the perspectives of a small sample of older Canadians and as such may not be readily transferable to other settings. We recognize this as a limitation and that our findings only reflect the perspectives of the 20 interviewees. Future research with larger samples of older adults is warranted. Second, we recognize our sample is undoubtedly overrepresentative of individuals who have the interest, access, and privilege to engage in new technologies. Although we specifically sought out individuals from a range of cohorts, living arrangements, and ethnic groups, our recruitment strategies (which had to be mindful of social distancing) mostly connected us with privileged individuals who were already online, had access to email, and were able to complete a voluntary research interview (ie, they had the time and interest to do so and, at the very least, had a telephone). These advantages will be reflected in our results, and this body of literature would greatly benefit from more work with older adults who do not/cannot use technology to receive care. In the future, recruitment options that do not rely on newer technologies should be used to connect with individuals who are less tech-savvy (eg, radio, mail-based, and in-person recruitment).

### Future Directions

Falk [34] has argued that virtual care may reduce inequities for some older persons, such as those living in remote communities, but at the same time might exacerbate inequities through avoiding direct service to these regions. Future research, including that of this team, must actively reach out to support those older adults on the underrepresented side of the digital divide [35-37], particularly as the United Nations calls for all nations to close these digital divides [38]. As suggested by participants in this study, support strategies should target both older adults and providers; Chen et al [39] found that training geriatric care professionals on virtual care technologies prior to the pandemic helped ease the transition to virtual care. Multiple virtual care resources have been designed for older adults in Canada, including appointment checklists (eg, [5]) and supportive liaisons to help navigate particular technologies, as implemented at Women’s College Hospital [40]. Technology-based interventions can also improve access for marginalized groups with less technical experience by simplifying user interfaces and workflows on virtual care platforms to increase usability [40,41]. Efforts should be made to collaborate with older adults when designing and implementing such strategies in order to maximize their usefulness and relevance [40,42,43]. This can be accomplished by engaging older adults in designing technology and virtual care systems, training providers, and research/program evaluation (eg, through advisory committees, participatory research, codesign, etc) [40].

### Conclusion

The COVID-19 pandemic has been a major catalyst for the adoption of virtual care in Canada [6]. Our study confirmed that the potential benefits of virtual care for older adults are numerous; despite barriers to accessing virtual care, many older adults perceive benefits and are open to continued use of virtual care after the pandemic. Our study also found many limitations of virtual care, and a consensus that virtual care should be an addendum to the health care system, rather than its main delivery mechanism. These findings would thus call into question policies, such as the United Kingdom’s National Health Service (NHS) plan for digital-first primary care for every patient [44]. As we transition to a postpandemic world, older adults must be included in discussions on the design and implementation of virtual care options. Concerns related to privacy and confidentiality have been highlighted in other studies [6,45] but were not significantly present in our findings; this could be an explanation for why some older adults were less comfortable accessing virtual care.

In this study, we presented data from a small sample of older adults from Canada detailing their experiences with virtual care during the pandemic, their perceptions on the benefits and limitations of virtual care, and their willingness to engage in virtual care. Future dissemination of virtual care options should ensure that older adults’ views, preferences, and circumstances are considered and that accommodations are made for those whose use of virtual care is limited by disability or discomfort with the technology. The findings can also be used to inform future studies on the use of virtual care by older adults, as providers and patients continue to adapt to both the potential and pitfalls of this mode of care delivery.

## Appendix

Multimedia Appendix 1 Interview guide.

## Abbreviations

BIPOC: Black, Indigenous, and people of color

## Data Availability

Anonymized data sets generated or analyzed during the study are available from the corresponding author on reasonable request.
