# Supplementary material for: The Impact of COVID-19 on Older Adults’ Perceptions of Virtual Care: Qualitative Study
Source: JMIR Aging. 2022 Oct 20;5(4):e38546. doi: 10.2196/38546 (PMC9586256; doi:10.2196/38546)
Supplement: Multimedia Appendix 1 [file aging_v5i4e38546_app1.docx]

**Interview Guide**

*Understanding and supporting the social connections and digital connectivity of older Canadians during and after the COVID-19 pandemic*

**BASELINE INTERVIEW:**

**Introduction Script**: *Thank you for agreeing to participate today.*

*I am going to ask you some questions about your experiences during the COVID-19 pandemic and about your social relationships and technology use during this time. There are no right or wrong answers: we are interested to hear your experiences and thoughts.*

*Before we start I just want to remind you that taking part is completely voluntary. Let me know if you want to skip a question or if you want to stop the interview. Do you have any questions for me at this time?*

**Part 1- General Tech Use Questions:**

1. Please tell me about a typical day for you right now.

Probes for: routines, habits, and potentially who they are speaking with/connecting with and how

1. What are one or two examples of how the COVID-19 pandemic has impact you, overall? These could be positive or negative impacts.
2. Please describe for me how the COVID-19 pandemic has impacted your relationships. This can be any relationship, be it family, friends, neighbours, acquaintances.
3. Thinking about the past week or two, who do you communicate with in a typical day? In a typical week?

- Probe for mechanisms: During Outings, Telephone, In-Person, Video Calls, Online, Via Social Media, etc.
- Probe for how this has changed/if this is different from pre-pandemic times

1. How has your communications with others changed since the start of the pandemic?
2. Please describe for me how the COVID-19 pandemic has impacted your mental health. (Note: Consider moving to end of this section, later in interview, depending on rapport and perceived comfort of the participant this early in the conversation).
3. Please tell us how often you use these communication technologies: (daily, once a week or more, a few times a month, a few times a year, never). After each technology, **where it seems appropriate,** ask about comfort, experience, how they learned to use, etc.

- TV
- Landline telephone
- Mobile/cellular phone
- Desktop computer
- Laptop computer
- Tablet (e.g., iPad or Microsoft Surface)
- Radio
- Other (please specify)

1. Are there any technologies that you would like to learn more about, or would like to try out?

- Probe for where did you hear about this? What has stopped you, so far, from trying it?
- In an era of social distancing, how would you like to/be able to learn to use X?

1. (Where appropriate) You seem pretty tech savvy/comfortable with technology. Why do you think that is? How did that come to be?

**Part 2- Virtual Care Questions:**

1. In this era of social distancing, many health care providers have introduced new technologies, like online or telephone appointments or, for example, sharing a photo of a rash over a secure app. These sorts of health care technologies we tend to refer to as “virtual care”, so things like telephone appointments, online appointments, perhaps sharing a photo of a condition over email, etc. Have your health care providers used any new technologies to support your care?

- If yes, can you tell me about this experience?

1. If your health care provider wanted to offer virtual health care services for older adults, what should that look like? Probes for: what mechanism (phone, virtual), what resources would you need, what training or supports would you need, etc.
2. What health professionals would you consider receiving virtual care from? (probe for family docs, nurses, pharmacists, physio, etc.)

Probes for what type of appointment (e.g., initial visit, follow-up, routine check-ins, etc.)

1. What do you think are the pros to virtual health care?
2. What do you think are the cons to virtual health care?
3. Would you prefer health care go "back to normal" after the pandemic, or can you picture virtual care being useful after the pandemic? What makes you say that? \
4. Thinking about yourself, and in particular others who might be less comfortable with technology, what are the barriers to virtual care for older adults?
5. What could we do to support older adults who want to receive virtual care?

**Demographic Questions:**

*I want to finish the interview by asking you a few questions about yourself. Please let me know if you prefer not to answer a question and we will go to the next one.*

What is your gender?

- *Female*
- *Male*
- *Self Identified ____________________________*

What is your year of birth?

What ethnic group(s) do you identify with?

Please tell me a little about your living arrangements.

-Probe for type of dwelling and number of people in household

Do you live in an urban, suburban, or rural area?

What province do you live in?

Do you have anything that you would like to add that we didn’t talk about today? Anything that you think might be relevant to this project or that you would like us to know?

**Complete closing statement & field notes.**

**FOLLOW-UP Interview:**

**Introduction Script**: *Thank you for agreeing to participate in a follow-up interview. It is so nice to connect with you again!*

*With your permission, we will be audio recording this session, as we did last time. This is a reminder that your name will not be associated with the data that you provide today. Do you agree to have this interview recorded?*

*Like last time, there are no right or wrong answers: we are interested to hear your experiences and thoughts about the ongoing COVID-19 pandemic.*

*Before we start, I just want to remind you that taking part is completely voluntary. Let me know if you want to skip a question or if you want to stop the interview. Do you have any questions for me at this time?*

**Part 1- Follow-up General Pandemic Questions:**

1. Last time we spoke, in the [late spring/early summer], we talked a bit about your social connections and relationships during the COVID-19 pandemic, and your daily routines. What does a typical day look like for you now?

Has that changed since we last spoke? If so, how?

2. What gets you up and going in the morning? What motivates you or gives your purpose in your daily life?

Follow-up: How has the pandemic impacted this purpose/role/motivation?

3. Please describe for me how the COVID-19 pandemic has impacted your relationships - this can be any relationship, be it family, friends, neighbours, acquaintances.

4. What was the hardest moment or era for you over the past year?

5. What positives have come out of your experience with this pandemic?

Prober for if/how any technologies have supported them in this .

6. What has helped you get through some of the challenges of the pandemic?

Probe for if/how any technologies supported them in this.

7. Some people have felt inspired or have felt like they have needed to start using new technologies to stay informed and connected during this pandemic. Have you started using any new technologies since we last spoke? This could be new devices, like a smartphone, new apps, or new tools?

If yes, probe for: what made you decided to start using X? What your experience like using that new technology?

If no, why haven’t you adopted any new technologies? What keeps you connected and informed?

***[Interviewer ALSO to follow-up on specific questions/topics raised in baseline interview]***

***Notes from baseline interview to follow-up on:***

**Part 2- Virtual care questions:**

8. In this era of social distancing, many health care providers have introduced new technologies, like online or telephone appointments or, for example, sharing a photo of a rash over a secure app. Have your health care providers used any new technologies to support your care?

-If yes, can you tell me about this experience?

- If no, would you be willing to try something like this if you had the right technologies and supports? What would you need to try this?

9. How confident are you in your provider’s ability to deliver care virtually? Is there any care-related activity that you believe will be less effective virtually (e.g. physical examination)?

10. How would you describe your relationship with your regular provider (e.g. family doctor)? How has it changed since transitioning to virtual visits/since the pandemic started?

11. Have you had a referral/transfer of information between providers during the pandemic? Can you describe your experience (e.g imaging referral, prescription)?

12. If your health care provider wanted to offer virtual health care services for older adults, what should that look like? Probes for: what mechanism (phone, virtual), what resources would you need, what training or supports would you need, etc.

- Are you more comfortable with audio or video conferencing?

13. What do you miss most about in-person visits/appointments? How can we provide something similar virtually?

14. What could we do to support older adults who want to receive virtual care?

Do you have anything that you would like to add that we didn’t talk about today? Anything that you think might be relevant to this project or that you would like us to know?

**Complete closing statement & field notes.**
